# Predictors of rehospitalization in Psychotic Patients after their first hospitalization

**DOI:** 10.1192/j.eurpsy.2022.1740

**Published:** 2022-09-01

**Authors:** P. Álvarez, A. Palau, C. Russo, E. Nieto

**Affiliations:** 1 Fundació Althaia Xarxa Assistencial of Manresa, Psychiatry, Manresa, Spain; 2 Fundaciò Althaia Xarxa Assistencial of Manresa, Psychiatry, Manresa (Barcelona), Spain

**Keywords:** hospitalization, paliperidone, psychotics, predictors

## Abstract

**Introduction:**

It is important to determine those clinical factors that imply a greater risk of rehospitalization in psychotic patients

**Objectives:**

To determine the rate and predictors of rehospitalization in psychotic patients after their first hospitalization

**Methods:**

We include all Psychotic patients admitted for first time in their life in our Psychiatric Unit between 2009 and 2019 (N=359) , including all diagnosed according DSM-IV of Schizophrenia or other Psychotic disorders -Multiple clinical, sociodemographic and biological variables of the basal hospitalization were recorded With the SPSS program we compared the variables between patients who needed any hospitalization in the follow-up until 31th December 2019 and those who do not. We use the Chi square ( qualitative variables) and the Student T ( quantitative variables)

**Results:**

109 psychotic inpatients had at least one rehospitalizations (30,4%). The qualitative variables significantly associated with rehospitalization were : cannabis in urine at admission (P<0.03), and treatment with risperidone (P<0.014). Instead treatment with long acting paliperidone was associated with absence of rehospitalization (P<0.005) .The quantitative variables relationed significantly with multiple rehospitalization were : lower age (P<0,015) lower HDL cholesterol levels (P<0.02) and higher years of follow-up after discharge (P<0.000)

**Conclusions:**

1-More of 30% of psychotic patients need rehospitalization after their first hospitalization in a mean of follow up of 5,8 years 2-Lower age, longer follow-up period and treatment with risperidone are significantly associated with rehospitalization , instead treatment with long acting paliperidone are significantly associated with absence of rehospitalization

**Disclosure:**

No significant relationships.

